# Monocular diplopia‐associated migraine‐like headache induced by nicotine withdrawal

**DOI:** 10.1002/ccr3.886

**Published:** 2017-03-17

**Authors:** Xiaoping He, Alvina Munaf, Evgeny Idrisov, George Everett, Sami K. Saikaly, Esther Kim, Khalid Abusaada, Vincent Hsu

**Affiliations:** ^1^ Internal Medicine Residency program of Florida HospitalOrlando, FloridaUSA; ^2^ UCF College of MedicineOrlando, FloridaUSA

**Keywords:** Diplopia, migraine, nicotine withdrawal

## Abstract

We describe an extremely rare case of migraine‐associated monocular diplopia developed in a 23‐year‐old man after sudden cessation of smoking. The physical examination and brain MRI scan were unremarkable. The symptoms resolved after starting nicotine patch. We reviewed the literature and discussed the diagnosis and possible mechanism of this phenomenon.

## Introduction

Diplopia is the visualization of an object in two different spatial locations. The causes of diplopia can range from simple benign refractive error to life threatening intracranial aneurysm 1. Diplopia generally represents a failure of binocular fusion of vision with the double image caused by a different location of the retinal image in either eye 2. Such diplopia is binocular and clears when monocular vision is used and only one retinal image is seen 2. Diplopia can also occur during use of only one eye (monocular diplopia), when two images are viewed by a single eye. Monocular diplopia is uncommon and always presents a diagnostic challenge 1. This report concerns a patient with monocular diplopia associated with nicotine withdrawal induced migraine headache.

## Case History/Examination

A 23‐year‐old nonhypertensive, nondiabetic African American male presented with a one‐week history of headache. His past medical history was significant for sickle cell disease which was in remission. His last sickle cell pain crisis occurred 5 years previously and currently he was not on any medication.

He was a half pack per day smoker for 7 years. Three weeks previously, he tried to quit smoking and began using a 14‐mg nicotine patch every day. He continued his smoking while he was on nicotine patch. One week later, he stopped both smoking and nicotine patch simultaneously. Two days later, he exhibited severe headache. The patient described the headache as moderate, constant, throbbing and sharp pain, encompassing the whole head. There were associated nausea, vomiting, phonophobia, sweats as well as decreased concentration and performance at work. The patient also reported intermittent double vision which started a few hours before he came to the hospital with each episode lasting no more than 5 min.

The general physical examination was unremarkable. Diplopia occurred during the interview, lasting no more than 5 min. Diplopia resolved when his right eye was covered, but remained when his left eye was covered. Ophthalmologic examination indicated the pupils were equal, round, and reactive to light. The extraocular movements and accommodation reflex were intact. Visual acuity was 20/20 in each eye at distance and near viewing. The vision field was normal on confrontation visual field examination. No papilledema was appreciated on fundoscopic examination. Neurologic examination was normal. The CBC, platelet count, coagulation studies, blood chemistry, thyroid function test, urinalysis, EKG, and chest X‐ray were normal. Urine toxicology screen was negative. MRI scan of the brain showed no abnormalities.

## Outcome and Follow‐up

The patient was given a 14‐mg nicotine patch and oxycodone for pain. All symptoms resolved within 1 day. The patient was educated regarding how to taper the nicotine patch and discharged. He has since reported no further episodes of monocular diplopia or migraine headache.

## Discussion

Although smoking has been considered by many people as a trigger of migraines headache 3, abstinence from smoking has also been reported to cause headaches. Upon smoking cessation, nicotine withdrawal symptoms can occur, peaking within the first 3 days 4. Headache is one of the most common symptoms of nicotine withdrawal with incidence of about 5–10% 5, 6. In this case, the patient developed typical migraine symptoms 2 days after the sudden discontinuation of a very high level of nicotine use and the symptoms quickly resolved after resuming nicotine patch, strongly suggesting that nicotine withdrawal was the cause of this episode of migraine. Although nicotine's pharmacologic aspects are extensively studied, the mechanism of nicotine withdrawal induced headache is not completely understood. There is accumulating evidence strongly implicating nicotine in the process of mediating pain. Nicotine can regulate neuronal activity by acting on the cys‐loop cation‐conducting ligand‐gated nicotinic ACh receptor channels (nAChRs), which are widely distributed throughout the central nervous system and participate in a variety of physiological responses such as anxiety, the central processing of pain, food intake, nicotine seeking behavior, and cognitive functions 7. The microinjection of nicotine into different regions of the brainstem produced an antinociceptive effect 8. Given the release of endogenous opioid beta‐endorphin by tobacco smoking, Schmidt et al. studied the role of nicotinic receptors (NRs) in the nucleus accumbens (NAs) where activity of opioids has been implicated in pain modulation. They found that intra‐NA NR blockade precipitated significant hyperalgesia in nicotine tolerant rats 9. It is reasonable to postulate that sudden discontinuation of nicotine use in our case may have resulted in rapid reversal of the analgesic effect following the acute exposure of high dosage of nicotine, thus leading to hyperalgesia which triggered migraine headache.

Of the migraine accompaniments, none is more puzzling than diplopia 10. Diplopia‐associated migraine is often classified under the subtype of ophthalmoplegic migraines (OM) which has been renamed “recurrent painful ophthalmoplegic neuropathy” (RPON) in the third edition of the International Classification of Headache Disorders (ICHD) 11. OM or RPON is a rare disorder, with a prevalence of 0.7 per one million 12. It typically starts in childhood, but adult onset cases have also been reported 13. Hallmarks of this disorder are defined as “recurrent attacks of headache with migrainous characteristics associated with paresis of one or more ocular cranial nerves (commonly the third nerve) in the absence of any demonstrable intracranial lesion other than MRI changes within the affected nerve 12.” Although most previous reports have not distinguished between monocular and binocular diplopia in OM, diplopia in OM generally represents a failure of binocular fusion of vision secondary to dysfunction of ocular motor nerve, which is typically binocular viewing.

To distinguish monocular versus binocular diplopia, the first step is to ask the patient to cover each eye separately. In the setting of binocular double vision, one of the two images disappears when either eye is covered 14. However, in our case, double vision resolved when the right eye was covered, but remained when the left eye was shielded, which clearly demonstrated the two images were viewed by single right eye (monocular diplopia). Moreover, in ophthalmoplegic migraine, the oculomotor nerve is affected most frequently. Pupillomotor fibers usually are involved, with resulting mydriasis and poor light response 10. Ptosis is also common. Specific examination features such as limitation of inward, outward, upward, or downward gaze depend on whether cranial nerve 3, 6, or 4 is involved. In our case, the pupils and eye movements were intact, suggesting no ocular nerves were affected. Negative urine toxicology screen excluded other drug abuse and brain MRI scan excluded the possible pain crisis. The diagnosis of monocular diplopia‐associated migraine was established. To the best of our knowledge, monocular diplopia‐associated migraine is extremely rare with only one pertinent publication available through a literature search of MEDLINE/PubMed, in which Dr. Drake reported a patient with bilateral monocular diplopia occurring during the attack of migraine 2. As in our case, no ocular disease was demonstrated and there was no indication of a potential neurologic cause for diplopia other than migraine.

Monocular diplopia can result from three conditions 1: light diffraction, retina pathology, and cerebral polyopia. Light diffraction may occur on the basis of ocular disease in the involved eye, chiefly cataracts, corneal opacity, astigmatism of severe degree, and dislocation of the lens, which cause images of a single object to fall on the fovea and the extrafoveal retina of the same eye. The images are of different clarity, with the extrafoveal ghost image overlapping the clear foveal image. Ophthalmology referral is indicated in this condition. Retina pathology can also cause monocular diplopia. Bielschowsky described a patient with long‐standing convergent strabismus and amblyopia of the left eye, who developed monocular diplopia in that eye after loss of the right eye 15. This was ascribed to divergence between the true macula of the left eye and the previous false macula in that amblyopic eye. Similarly, Cass reported monocular diplopia in 33 of 70 patients with strabismus 15. Cerebral diplopia or polyopia describes the perception of multiple images and arises from an occipital disturbance. Zakaria reported a case of a patient with monocular polyopia as the primary manifestation of a simple partial occipital lobe seizure 16. Monocular diplopia attributable to a localized occipital lobe infarct was also described 17. Cerebral diplopia can occur with migraine headaches 2. After ruling out the above two other reasons, cerebral diplopia might be the contributor to monocular diplopia in migraine attack 2. Given that monocular diplopia in our case was transient, intermittent, and fully recovered without any residual neurologic deficits, it was more often of functional origin.

There are several different characteristics between monocular and binocular diplopia occurring with migraine. First, In OM, the headaches usually precede the ophthalmoplegia by 3–4 days and resolve quickly. Binocular diplopia may develop during or after the headache phase 11, while monocular diplopia usually develops during the headache phase. Second, the duration of diplopia in OM varies. In the majority of the reported cases, binocular diplopia resolves over days to weeks or even months and residual deficits of the oculomotor nerve may occur from repeated episodes gone untreated 11. Monocular diplopia usually disappears after headache resolves. Third, recently OM has been classified as a demyelinating condition affecting the oculomotor nerves with MRI changes within the affected nerve 12. The oculomotor nerve is most frequently affected, followed by the abducens 12 and in rare cases the trochlear nerve 10. Monocular diplopia in migraine secondary to cerebral diplopia is more likely to have a possible microvascular, ischemic etiology with normal brain MRI.

In summary, we reported a rare case of monocular diplopia associated with nicotine‐withdraw induced migraine. It is important to distinguish monocular versus binocular diplopia in migraine because it may represent a different disease entity. The prognosis under either condition is generally good, as both headache and diplopia resolve spontaneously. In addition, tapering the use of nicotine patch is recommended during cessation of smoking to avoid withdrawal symptoms.

## Authorship

All those involved have read and approved the final manuscript. HX, MA, IE, SS, KE: drafted the manuscript. EG, AK, HV: revised the manuscript critically for important intellectual content. HX, MA, EG, SS, and KE: treated the patient described in the manuscript.

## Conflict of Interest

None declared.
